# Conference at Central Hall, Westminster

**Published:** 1919-05-10

**Authors:** 


### THE MEDICAL PARLIAMENTARY COMMITTEE.

### Conference at Central Hall, Westminster.

A CONFERENCE called by the Medical Parliamentary Committee, and attended by various medical, nursing, and pharmaceutical societies' representatives, was held at the Central Hall, Westminster, on May 2, under the presidency of Sir Watson Cheyne, C.B., M.S., M.P. The purpose of the Conference was to set up a Medical Parliamentary Committee on a permanent basis.

Sir WATSON CHEYNE said that the whole question had arisen from the fact that the medical profession did not get sufficient importance attached to its opinions in legislation and public matters.

Dr. ARTHUR LATHAM proposed the first resolution: "That a permanent Medical Parliamentary Committee be formed." He said they had to devise some efficient machinery for ascertaining the views of the profession, and for ventilating those views before Parliament and the public. Hitherto the medical profession had had the habit, so far as politics were concerned, of "losing the train." As to using an existing organisation for the purpose indicated, he was not satisfied that any such organisation could afford the time or possessed adequate machinery for the purpose in view.

Sir HENRY MORRIS, in seconding, hoped the British Medical Association would be represented on the Committee. The function of the Committee, he thought, should be not only to furnish information to medical members of Parliament as to the trend of expert opinion, but also to give information to members of the Government and members of Parliament generally. Incidentally, he complained of the fact that on so important a matter as the Dogs' Protection Bill there was not a single speech made by a medical member of Parliament when that measure came up for second reading. He was aware, however, that the Bill was brought on by one of the greatest experts in Parliamentary tactics, and he had no doubt that he had stolen a march upon the medical members of Parliament, most of whom were comparatively new to Parliamentary life.

Dr. H. B. MORGAN moved an amendment recommending the British Medical Association to co-opt on its Medico-Political Committee elected representatives of other national organisations, so as to have a collective medical opinion under the Association's auspices. The CHAIRMAN refused to accept the amendment, and Dr. MORGAN thereupon contented himself with an attack upon the Medical Parliamentary Committee, whom he described as a body of consultants, representing only themselves.

On the motion being put to the meeting, it was carried with one or two dissentients.—Colonel FARQUHARSON, M.P., raised the question of the vote-value at that Conference. Some, he said, were present representing only themselves, while others represented large organisations. The CHAIRMAN, however, ruled that votes should have an equal standing, because those who represented associations on that occasion were not pledging such associations; the gathering was purely a Conference.

A long discussion ensued on the first suggested function of the Medical Parliamentary Committee, which was given on the agenda as that of "Supplying information of the trend of expert opinion on health questions to medical members of Parliament." Ultimately the clause was generalised into "Supplying information on the trend of opinion on health questions," and in this form was carried.

Sir T. JENNER VERRALL, who represented the British Medical Association, said that he could not pledge his Association for or against the adoption of the scheme for a Medical Parliamentary Committee. The Council of the Association would no doubt consider the matter and report upon it to the members generally, but it might easily be that the Association when the matter was put before it would decide—and, indeed, would be bound to decide—that the proper way to construct a Medical Parliamentary Committee would be to do it itself, with or without co-option of other bodies outside. The British Medical Association already performed the functions laid down as expressing the purpose for which the Medical Parliamentary Committee should be formed (supplying information of the trend of opinion on health questions; supplying early information of impending legislation to the constituent bodies; facilitating communication between the several bodies affected by the proposed legislation, and assisting in increasing medical representation in Parliament). If it were left to the Association to construct a permanent Parliamentary Committee, he believed that such a Committee would be set up as would command the confidence of all the various bodies concerned—the nursing profession, the pharmacists, the hospitals, etc. He claimed that the British Medical Association was a very democratic body, and that if it were unrepresentative in any particular it was always open to reconstruction.

Dr. E. H. M. STANCOMB said that the main function of the proposed Committee was to gain political power for the medical men of this country. The primordial and fundamental industry of the country was that of health, and it had got no representatives in Parliament—that is to say, none who were sent there as medical men. There were medical men in Parliament, but they did not get into Parliament as doctors; they went there under the party system.—Three members of Parliament who were in the Conference (Sir William Whitla, Dr. Nathan Raw, and Col. Farquharson) strongly objected to this statement, and Dr. Stancomb accepted their disclaimer, but he said that they had got in under party organisation more or less. (Sir W. Whitla: "No.") Dr. Stancomb said that he was making it his aim to sit in Parliament as a representative of national health and nothing else.

The clauses indicating the purposes of the proposed Committee (given above) were then carried, and Dr. C. BUTTAR moved a comprehensive resolution determining the constitution of the Committee. He said that at present the idea was to get representatives of various bodies rather than to have representatives elected on a territorial basis, though this might come later. These bodies included associations other than those of medical men, and he defended that course of action. Sir W. WHITLA asked whether any representative of Ireland was included. Dr. MAGENNIS said that he, as representing the Irish Medical Association, supported this Medical Parliamentary Committee. Dr. JANE WALKER seconded the motion determining the constitution of the Committee. Dr. V. T. GREENYER wanted to move that the Committee be composed of medical practitioners only, and that a Sub-Committee consist of these ancillary non-medical bodies, but he did not press his motion. Dr. E. G. NEWALL proposed certain other inclusions, but these were rejected. As finally carried, the Committee will consist of a representative or representatives of any society or association of registered medical or dental practitioners; two representatives of the nursing profession, one each of the midwives, the pharmaceutical societies, the British Hospitals' Association, and the Housing and Town Planning Association; the editors of the *British Medical Journal, Lancet, Medical Press and Circular, Medical World,* and *Medical Times*; all the medical members of Parliament; co-opted members, not exceeding one-fifth of the Committee, of whom not more than a quarter should be engaged in consulting practice; and directly elected representatives of the profession, to be elected as soon as the necessary expenditure was warranted.

In reply to Mr. E. B. Turner, Dr. LATHAM said that to have a representative in the lobby and a good secretariat would mean an expenditure of between £1,500 and £2,000 a year. The money could be found for the first few months (already some £700 subscribed privately had been spent), and after that the Committee must stand or fall by its achievements. It was agreed, on the motion of the CHAIRMAN, that the Committee meet at least twice a year, that it appoint an executive, which should meet at least once a month, and have power to appoint Sub-Committees.

